# Empowering adolescent girls: developing egalitarian gender norms and relations to end violence

**DOI:** 10.1186/1742-4755-11-75

**Published:** 2014-10-21

**Authors:** Avni Amin, Venkatraman Chandra-Mouli

**Affiliations:** Department for Reproductive Health Research, World Health Organization, Avenue Appia 20, 1211 Geneva 27, Switzerland

## Abstract

On the occasion of the International Day of the Girl Child (October 11), this commentary highlights the problem of violence against adolescent girls. It describes the nature and magnitude of violence faced by adolescent girls, what we know about factors that drive violence against women and against adolescent girls. It highlights the importance of promoting egalitarian gender norms, particularly during adolescence, and empowering women and girls in efforts to end such violence. Finally, it offers lessons learned from some promising interventions in this area.

The theme of this year’s (2014) United Nations International Day of the Girl Child is: *Empowering adolescent girls: Ending the cycle of violence*. Violence against women and against girls is a widespread public health problem, a violation of human rights and one that is rooted in gender inequality. This paper describes what we know about factors that drive violence against women and against adolescent girls. It highlights the importance of promoting egalitarian gender norms and empowering women and girls in efforts to end the cycle of such violence. It also offers lessons learned from some promising interventions in this area.

Adolescent girls (10–19 years) face different forms of violence depending on their age and also depending on whether they are married or have partners (casual, or long-term) or not. Both adolescent girls and boys are subjected to physical and emotional abuse by those who have power and authority over them (e.g. parents, caregivers, guardians, teachers) and by peers. They also experience other forms of violence such as bullying. Adolescent girls also face specific forms of violence that are related to gender inequality such as a higher risk of sexual violence compared to boys, trafficking, and, in some regions, female genital mutilation, early and forced marriage, and murders in the name of honour. In addition, girls who are married or are in dating relationships face a high risk of intimate partner violence.

Globally, 1 in 3 women will experience physical and/or sexual violence by an intimate partner or sexual violence by someone other than their partner. Such violence starts early in the lives of women, with estimates showing that nearly 30% of adolescent girls (15–19 years) have experienced intimate partner violence [1]. Data from 21 countries highlight that among girls (15–19 years) who had experienced sexual violence, a significant proportion of them had experienced sexual violence for the first time before 15 years of age. Data from 17 countries show that between 1 and 29% of women and girls (15–49 years) reported that their first experience of sex was coerced, with a higher likelihood of experiencing coerced sex among those who had their first experience of sex when they were between 15–19 years old [2]. Such violence has serious and often life-long consequences for adolescent girls as they transition into adulthood ranging from physical injuries to unintended pregnancies, adverse maternal and child health outcomes (e.g. pregnancy loss, low birth weight babies), sexually transmitted infections including HIV and poor mental health outcomes (e.g. depression, post-traumatic stress disorder) [1].

The risk factors for violence against women and against girls vary depending on the type of violence and on the context. Risk factors for women’s experience of intimate partner violence include: exposure to violence during childhood; harmful use of alcohol and substance abuse; controlling male behavior in intimate relationships; unequal gender norms including those that condone violence against women and girls; and laws and policies that perpetuate gender inequality [3, 4]. Studies show that women’s attitudes justifying a man beating his wife (a proxy for norms tolerant of violence) is a significant risk factor for their experience of intimate partner violence [3]. Research also shows that men and boys who are exposed to childhood violence and who hold unequal gender attitudes are more likely to perpetrate violence against women and girls [5].

The gender attitudes of both women and men are shaped by peers, family members, others in the community including community leaders, and societal institutions (e.g. media, sports, religious, military, schools) that validate masculine norms and identities and female subordination [6]. Gender socialization starts in early childhood when boys and girls are treated differently and given gender specific toys and messages (e.g. boys don’t cry and girls must behave ‘lady-like’). Population-based surveys from 10 countries show that rigid gender attitudes begin to form early in the lives of adolescents, with 50-83% of boys (15–19 years) reporting that it is justifiable for a man to beat his wife under certain circumstances [7]. Adolescence is a crucial period when boys and girls go through puberty related changes, explore their sexuality, further develop their gender identities, attitudes and behaviors, and may begin to form intimate relationships. As such, it provides a critical opportunity to shape non-violent and egalitarian attitudes and norms and healthy sexual and reproductive health behaviors before these become rigid and entrenched.

Twenty years ago, the Cairo International Conference on Population and Development (1994) Programme of Action shifted the paradigm of population control to a rights-based approach to sexual and reproductive health [8]. It recognized the importance of promoting gender equality and addressing gender-based violence including violence against adolescent girls as critical to these efforts [8]. Since then, a number of agencies including the United Nations, donors and many community-based and international NGOs have undertaken programmes and interventions to promote gender equality including in the context of sexual and reproductive health programmes and as part of efforts to address violence against women and against girls.

There is emerging evidence of what works to promote, change and sustain egalitarian gender norms and attitudes and behaviors among adolescent girls and boys as they transition into adulthood. For example, there are increasing efforts to implement programmes to empower girls and young women to prevent or reduce violence and to promote their sexual and reproductive health. A small number of them that have been rigorously evaluated include: a) a conditional cash transfer intervention to keep girls in school in Malawi that showed reductions in HIV prevalence and in unintended pregnancies (but did not consider violence as an outcome); and b) a combined livelihood and life-skills education intervention in Zimbabwe for adolescent girls (16–19 years) that showed a 58% decrease in personal experience of physical and sexual violence over two years, an increase in equitable gender attitudes and a decrease in food insecurity among programme participants [9–11].

There are also several programmes being implemented to challenge unequal and harmful gender norms and attitudes. These interventions have either worked with groups of boys and men and/or girls and women or with entire communities. A small number of them have been rigorously evaluated. These include: a) small group community-based participatory education in South Africa with young men and women (15–26 years) to foster critical reflection on gender norms and relations that showed a 38% reduction in men’s perpetration of violence after 2 years of intervention [12]; b) individual and small group school interventions (India) and community-based participatory education with men and boys and adolescents (boys and girls) combined with mass media campaigns (e.g. soap operas, edutainment, lifestyle campaigns) in Brazil, India, Ethiopia and Nicaragua to challenge masculine and other gender norms that showed improvements in attitudes towards gender equality and towards acceptability of violence against women, but were less successful in changing behaviours including perpetration of violence [13–17]; and c) a community-mobilization intervention in Uganda that sought to generate critical reflection among all members of the community on unequal gender power dynamics that showed a 46% and 87% reduction among women and men respectively in social acceptance of violence against women, and a 52% and 34% reduction in physical and sexual partner violence respectively among intervention participants [18].

The evidence base on what works to promote empowerment of adolescent girls and egalitarian gender attitudes, norms and behaviors among adolescents (both girls and boys) needs strengthening. First, programmes need to be evaluated and with stronger designs (e.g. experimental designs with individual or cluster randomization). Second, outcomes need to go beyond measuring individual attitudes to changes in community level norms and in behaviors. And lastly, evaluations need to consider sustained behavior changes over time beyond the typical 6 to 12 months post-interventions that most studies have done so far.

There are a few important lessons learned for promoting gender equality and empowerment of girls and women. First, there is an emerging consensus that it is no longer enough to work only with girls or only with boys. The literature on gender equality has been polarized by pitching a women and girls only approach versus an approach focusing on men and boys [19, 20]. Research shows that success is more likely where interventions have worked with both, boys and girls, men and women in a synergistic or synchronized manner. While there has been considerable emphasis on challenging “dominant masculinities”, there is an equally critical need to challenge “passive femininities” or norms that perpetuate female subordination and have devastating impacts on girls’ self-esteem, body image, and their ability to assert themselves in their relationships. And lastly, challenging harmful gender norms (both masculine and feminine) and unequal power between women and men and boys and girls requires going beyond individual level efforts (i.e. working with individuals or groups of girls or boys) to challenging gender inequalities at the structural level. Specifically, this requires implementing strategies with whole communities (e.g. community and religious leaders, parents, family members, peers) and institutions (e.g. schools, sports, media, religious, health, law enforcement, justice, political) to support and sustain widespread societal changes in harmful gender norms and in discriminatory practices in order to end violence against girls and women and improve their health and wellbeing.
